# Caveolin as a Novel Potential Therapeutic Target in Cardiac and Vascular Diseases: A Mini Review

**DOI:** 10.14336/AD.2019.09603

**Published:** 2020-03-09

**Authors:** Jinfan Tian, Mohammad Sharif Popal, RongChong Huang, Min Zhang, Xin Zhao, Mingduo Zhang, Xiantao Song

**Affiliations:** ^1^ Department of Cardiology, Beijing Anzhen Hospital, Capital Medical University, Beijing 100029, China.; ^2^ Department of Cardiac Surgery, Beijing Anzhen Hospital, Capital Medical University, Beijing 100029, China.; ^3^ Department of Cardiology, Beijing Friendship Hospital, Capital Medical University, Beijing 100010, China.

**Keywords:** Caveolin, autophagy, cardiovascular disease, role, target

## Abstract

Caveolin, a structural protein of caveolae, play roles in the regulation of endothelial function, cellular lipid homeostasis, and cardiac function by affecting the activity and biogenesis of nitric oxide, and by modulating signal transduction pathways that mediate inflammatory responses and oxidative stress. In this review, we present the role of caveolin in cardiac and vascular diseases and the relevant signaling pathways involved. Furthermore, we discuss a novel therapeutic perspective comprising crosstalk between caveolin and autophagy.

## 1. Introduction

Caveolae are characterized as 50 to 100-nm invaginations of the cell-surface plasma-membrane; initially described by Palade and Yamada, caveolae are a specialized form of lipid rafts found in differentiated cell types including endothelial cells and vascular smooth muscle cells [1]. Caveolae are abundantly present in ventricular, atrial, and nodal cells. They are rich in sphingolipids, cholesterol, and lipid-anchored proteins. Caveolin, the main structural protein of caveolae, is essential for caveolae formation and for distinguish caveolae from lipid rafts. Cavins, which were recently identified to be localized caveolae, are important for caveolar biogenesis, caveolin expression, and caveolae morphology [2, 3]. There are three main isoforms of caveolin, namely caveolin-1, caveolin-2, and caveolin-3 [4]. Caveolin-1 and caveolin-2 are generally expressed in smooth muscle cells, endothelial cells, skeletal myoblasts, fibroblasts, and adipocytes. Caveolin-3 is primarily expressed in skeletal, cardiac, and vascular smooth muscle cells. Caveolins are hairpin-shaped proteins embedded in the cytosolic leaflet of the plasma membrane, with both N- and C-termini residing in the cytosol. The scaffolding domain of caveolin binds to pro-survival and pro-growth molecules, such as endothelial nitric synthase (eNOS), p42/p44 mitogen-activated protein kinase (MAPK), Akt, PKA, PKC, SFK, and glycogen synthase kinase-3β, which enable caveolin to regulate signaling pathways such as G protein-coupled receptor, tyrosine kinases, eNOS, and MAPK pathways, which are associated with inflammation and phonotypic changes in the vascular wall and myocardium [5, 6]. Recently, caveolin was reported to prevent NO release; moreover, it is closely related to changes in vascular tone change as it binds eNOS or inhibits the activity of this protein by limiting its accessibility to calcium-calmodulin binding. Consequently, it has been established that caveolin plays a crucial role in cardiovascular diseases including atherosclerosis, coronary microcirculation dysfunction, cardiomyopathy, heart failure, ischemic reperfusion injury, pulmonary hypertension, and ischemic stroke. Because various different ion channels are localized to caveolae, caveolar ion channels are implicated in inherited arrhythmias such as long QT Syndrome 3 (LQT3) and long QT Syndrome 9 (LQT9) and in acquired arrhythmias that result from heart failure [7].

Therefore, in this review, we present the role of caveolin in cardiac and vascular diseases and the relevant signaling pathways involved, as well as discuss a novel therapeutic perspective of a crosstalk between caveolin and autophagy.

## 2. Caveolin and atherosclerosis

**Figure 1.**
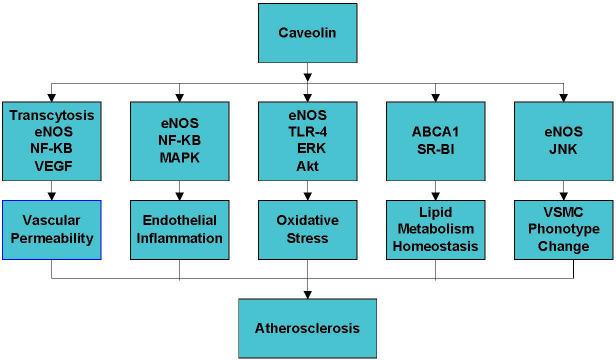
The mechanisms of the role of caveolin in atherosclerosis. Caveolin plays a critical role in atherosclerosis by regulating vascular permeability, endothelial inflammation, oxidative stress and lipid metabolism homeostasis.

Caveolin is essential for various cellular processes such as endocytosis, cholesterol homeostasis, inflammation, oxidative stress, and vascular smooth muscle cell proliferation [8-11] (Fig. 1). The effect of caveolin on atherosclerosis mainly depends on the cell type and metabolic pathways regulated by this protein [11, 12].

### 2.1 The role of caveolin in endothelial dysfunction and associated signaling pathways

More than 50 years ago, caveolae were reported to be present in endothelial cells. The endothelium plays a crucial role in regulating blood vessel function by modulating vascular tone, inflammation, oxidative stress, and hemodynamics. Endothelial dysfunction occurs upon stimulation of the endothelium by various pathological factors. Endothelial dysfunction, followed by the recruitment of macrophages, enhances the uptake of oxidized low-density lipoprotein (ox-LDL), disrupts vascular permeability, impairs endothelium-dependent vasodilatation, promotes endothelial inflammation and oxidative stress, and contributes to the progression of atherosclerosis.

Endothelial caveolin-1 plays a critical role in the initial step of atherosclerosis; it increases ox-LDL uptake through endocytosis, and potentially transcytosis across endothelial cells, and through the disruption of endothelial permeability [9]. Pavlides S et al. [9] reported that silencing caveolin-1 prevents LDL endocytosis, indicating a direct role for this protein in regulating LDL uptake. A recent study by Gerbod-Giannone MC et al. also revealed that caveolae and CD36 are involved in the endocytosis of native LDL [13]. Moreover, according to Sun et al. [14], the NF-kB pathway is mechanically involved in caveolin-1-promoted ox-LDL uptake by human umbilical vein endothelial cells (HUVECs). The capability of caveolin to transfer molecules from the lumen to the sub-endothelial space of blood vessels, as well as the inhibitory effect of caveolae/caveolin on eNOS, enable caveolin-1 to induce endothelial cell dysfunction by regulating vascular permeability [15]. Furthermore, several signaling pathways and molecules are involved in the regulation of vascular permeability by caveolin, including NF-kB and vascular endothelial growth factor.

Endothelial inflammation and oxidative stress induced by ox-LDL and macrophage migration contribute to endothelial cell apoptosis and atherosclerosis. Lin et al. [16] revealed that ox-LDL upregulates caveolin-1 phosphorylation, which leads to HUVEC apoptosis and macrophage recruitment, suggesting that caveolin is positively associated with inflammation. Caveolin also exerts inflammation-regulatory effects by modulating inflammatory signaling pathways including NF-kB and MAPK [17]. Further, caveolin directly interacts with and binds eNOS [6], Toll-like receptor (TLR-4), ERK, and Akt [4]. Moreover, under conditions of shear stress, caveolin can disassemble and uncouple from these signaling molecules due to imbalances in ROS and NO homeostasis, as well as subsequent activation of the endothelium, eventually leading to the enhanced transcriptional activity of pro-inflammatory molecules mediated by NF-kB and MAPKs [4, 5]. Pavlides S et al. [9] found that caveolin-1 regulates the TNF-α signaling pathway in endothelial cells, thereby regulating activation of NF-kB. Further, compared to that in ApoE^-/-^ mice, caveolin-1^-/-^ ApoE^-/-^ mice showed reduced endothelial activation and reduced VCAM-1 expression in the aorta, which was partly due to the disruptions in the transfer and accumulation of LDL in the arterial wall [18]. Moreover, the inhibition of caveolin-1 results in the attenuation of atherosclerotic lesions in ApoE^-/-^ mice, which is associated with decreased NF-kB-mediated inflammation [19]. Furthermore, caveolae have also been reported to regulate ROS production through NADPH oxidase (Nox) by modulating oxidative stress and ox-LDL-induced cellular senescence in the vasculature [4, 20]. These results suggest that endothelial caveolin-1 plays a proatherogenic role in the early stages of atherosclerosis development by enhancing LDL uptake, endothelial permeability, inflammation, and oxidative stress [9, 18, 21].

### 2.2 Role of caveolin in intracellular lipid metabolism homeostasis and macrophage scavenging

It has been established that impaired cholesterol efflux leads to the accumulation of intracellular cholesterol and the development of atherosclerosis. The effect of caveolin on intracellular cholesterol is controversial. Wang L et al. [22] revealed that caveolin-1 does not affect scavenger receptor class B member 1 (SR-BI)-mediated cholesterol efflux or the selective uptake of cholesteryl ester in Fischer rat thyroid cells and HEK 293 cells. Interestingly, Matveev S et al. [23] reported that the expression of caveolin-1 inhibits the SR-BI-dependent selective uptake of cholesteryl ester. Frank PG et al. [24-26] suggested that caveolin-1 plays an important role in regulating cellular cholesterol homeostasis, which might explain the diminished susceptibility to atherosclerosis in caveolin-1-deficient mice, as compared to that in wild-type mice, with an ApoE^-/-^ background. According to Frank PG et al., caveolin-1 regulates the expression of ATP-binding cassette transporter-1 (ABCA1); therefore, it might directly affect cellular cholesterol efflux to apolipoprotein A-I (apoA-I) [25]. Hu *et al.* [12] showed that the specific downregulation of caveolin-1 impairs cholesterol efflux to apo A-I. Moreover, PPAR γ-treated RAW264.7 cells administered caveolin-1 siRNA exhibited enhanced cholesterol efflux mediated by the PPARγ-LXRα-caveolin-1 or PPARγ-LXRα-ABCA1 pathway. Furthermore, another study also revealed that caveolin-1-knockout mice show lower ABCA1 expression in macrophages compared to that in the control group, suggesting that caveolin-1 is involved in regulating ABCA1-mediated cholesterol efflux [27]. Taken together, cholesterol efflux regulation is one of the mechanisms through which caveolin affects atherosclerosis.

### 2.3 Caveolin and phonotypic changes in smooth muscle cells

Caveolin plays a critical role in atherosclerosis by modulating inflammation or vascular remodeling in vascular smooth muscle cells. Wang et al. revealed that caveolin-1 promotes atherosclerosis in ApoE^-/-^ mice by upregulating ox-LDL-induced inflammation in vascular smooth muscle cells, which is mediated by JNK activation [17]. Similarly, Forrester SJ et al. [28] revealed that caveolin-1^+/+^ mice show increased AngII-induced vascular remodeling. In contrast, caveolin-1^-/-^ mice exhibit the attenuation of AngII-induced vascular remodeling. However, other studies revealed different roles of caveolin in regulating the remodeling of vascular smooth muscle. Zhou et al. [29] found that the knockdown of cavin-1 via the local injection of short hairpin RNA into balloon-injured carotid arteries in vivo promotes neointimal formation. Additionally, the inhibition of caveolin-1 in cultured vascular smooth muscle cells in vitro was found to promote the proliferation and migration of smooth muscle cells by increasing extracellular signal-regulated kinase phosphorylation and matrix-degrading metalloproteinase-9 (MMP9) activity. Moreover, Schwencke C et al. [30] showed that the adenoviral overexpression of caveolin-1 inhibits smooth muscle cell proliferation and that the expression of caveolin-1 in vivo is significantly decreased in proliferating vascular smooth cells of human atheroma, suggesting that the loss of antiproliferative control by caveolin-1 plays a pivotal role in vascular smooth muscle cell proliferation during atherosclerosis. Furthermore, Gutierrez-Pajares JL et al. [31] revealed that caveolin-3 promotes the contractile phenotype of vascular smooth muscle cells and reduces cell proliferation and migration, indicating that downregulating caveolin-3 contributes to atherosclerosis development or restenosis by promoting vascular dedifferentiation. Hence, modulating vascular smooth muscle remodeling is another mechanism through which caveolin regulates atherosclerosis.

## 3. Caveolin and coronary microvascular function

It has been shown that endothelium-dependent hyperpolarization (EDH) rather than NO plays a dominant role in small resistance vessels. The endothelium, which serves as a NO-generating system, is functionally inhibited in resistance vessels through a caveoin-1-dependent mechanism, switching its function from a NO-generating enzyme to an EDH/H2O2-generating enzyme in mice [32]. Caveolin-1-knockout and eNOS-Tg mice show a disrupted balance between NO and EDH during endothelium-dependent relaxation, as well as a reduced EDH-mediated coronary microcirculation response. In contrast, the reintroduction of caveolin-1 into the endothelium of caveolin-1-knockout mice was found to rescue the impaired EDH-mediated relaxation of small mesenteric arteries [33]. Hence, it was indicated that caveolin is a promising target to improve microvascular dysfunction.

## 4. Caveolin and occlusive coronary artery-related ischemic/reperfusion injury

It is considered that ischemic preconditioning can protect the heart from ischemia-reperfusion injury. According to Patel HH et al. [34], ischemic preconditioning increases the phosphorylation of caveolin-1. Further, disruptions in cardiac myocyte caveolae fully attenuate the protective effects of ischemic preconditioning [35]. Jasmin JF et al. [36] showed the role of caveolin-1 in myocardial ischemia-induced cardiac dysfunction, revealing that survival is lower in caveolin-1-knockout mice subjected to left descending artery ligation than in wild-type mice. Despite similar infarct sizes, caveolin-1-knockout mice subjected to myocardial infarction showed a decreased left ventricular ejection fraction and fractional shortening, as well as increased left-ventricular diastolic pressures, as compared to those in control mice. The mechanisms underlying these effects in caveolin-1-knockout mice subjected to myocardial infarction are the reduced density of β-adrenergic receptors at the plasma membrane and diminished cAMP levels and PKA phosphorylation. According to Kaakinen M et al. [37], hearts with deficiencies in caveolin-1 and caveolin-3 show decreased contractile dysfunction and cell damage following ischemia. In contrast, Tsutsumi YM et al. [38] revealed that mice overexpressing caveolin-3 subjected to ischemia/reperfusion injury show a significantly reduced infarct size. Further, the overexpression of caveolin-3 induces cardiac protection similar to that observed in wild-type mice undergoing ischemic preconditioning; mechanically, mice overexpressing caveolin-3 have increased basal Akt and GSK3β phosphorylation compared to those in wild-type mice exposed to ischemic preconditioning. Zhu et al. [39] showed that, in the context of ischemic/reperfusion, propofol pretreatment decreases the left ventricle infarct size in rats. In addition, the inhibition of caveolin-3 in vitro abolished propofol-induced cardiac protection and PI3K/Akt/GSK3β activation, indicating that caveolin-3 mediates propofol-induced cardioprotection against ischemic/reperfusion injury through PI3K/Akt/GSK3β activation. These findings showed that the overexpression of caveolin-1 and caveolin-3 protects against ischemic/reperfusion injury.

## 5. Caveolin and cardiac function

Cardiomyocytes mainly express caveolin-3. The major role of caveolin-1 in cardiac function is its function in supporting cells of the heart (fibroblasts and endothelial cells) [40]. Recently, the expression of caveolin-1 in H9c2 cardiomyocytes was reported [41]. Further, activation of the p42/44MAPK cascade (MEK1/2 and ERK1/2) has been shown to be closely associated with cardiac hypertrophy. Caveolin-1-knockout mice exhibit myocardial hypertrophy due to constitutive activation of the p42/44 MAPK pathway and hyperphosphorylation of ERK1/2 [42]. In contrast, cardiac hypertrophy was found to be reversed by the reconstitution of caveolin-1 [43]. These investigators showed that hyperactivation of the p42/44 MAPK pathway and hyperphosphorylation of ERK1/2 due to caveolion-1-knockout is confined to the areas of interstitial fibrosis and does not occur within cardiac myocytes. The hyperactivation of ERK1/2 due to caveolin-1 deficiency also occurs in isolated cardiac fibroblasts. Hence, it is hypothesized that caveolin-1, which is expressed in fibroblasts, exerts inhibitory effects on ERK1/2 activation [42]. Other studies revealed that in addition to ERK1/2 signaling, the negative regulation of eNOS by caveolin-1, which results in constitutive hyperactivation of the nitric oxide pathway in caveolin-1-knockout mice, is also involved in the development of severe cardiomyopathy and impaired pump function [43, 44]. Furthermore, the endothelium-specific reconstitution of caveolin-1 rescues the cardiac defects in global caveolin-1-knockout mice [45]. Wu et al. [41] also reported the role of caveolin-1 in H9c2 cardiomyocytes. They found that apelin, which was reported to be involved in cardiomyocyte hypertrophy, is significantly increased in rat models of left ventricular hypertrophy (LVH) established through constriction of the abdominal aorta. In contrast, caveolin-1 is decreased in LVH rats. The investigators further showed that in cultured H9c2 cardiomyocytes, caveolin-1 is suppressed by apelin-3, which is reversed by the apelin receptor F13A. Moreover, the overexpression of caveolin-1 reduces the diameter and volume of H9c2 cells. Consequently, the genetic disruption of caveolin-1 is sufficient to induce severe biventricular hypertrophy with signs of both systolic and diastolic heart failure [46].

The hearts with caveolin-3 knockout show significant hypertrophy, dilation, and reduced fractional shortening, as revealed by cardiac MRI and transthoracic echocardiography. Additionally, the loss of caveolin-3 induces significant cardiac myocyte hypertrophy, which is accompanied by cellular infiltration and progressive interstitial/peri-vascular fibrosis, as revealed by histological analysis. Activation of the p42/44 MAPK cascade, which is negatively regulated by caveolin-3, is associated with these changes [47]. In addition, other signaling pathways such as beta2-adrenergic receptor-mediated cAMP (β2AR-cAMP), PKC, and calcineurin/nuclear factor of activated T cell signaling (NFAT) pathways mediate the role of caveolin in the heart function. Further, the dominant-negative disruption of caveolin-3 function leads to far-reaching β2AR-cAMP signals similar to those observed in heart failure. Alternatively, the overexpression of caveolin-3 can partially restore the disrupted localization of these receptors in failing cardiomyocytes [48]. Bryant SM et al. also discussed that the regulation of L-type Ca^2+^ current by β2AR is redistributed to the surface membrane in failure heart caused by low of constitutive regulation by caveolin-3 [49]. Furthermore, the overexpression of caveolin-3 protects mice from transverse aortic constriction or Ang-II-induced pathological hypertrophy by preventing a PKC-α-mediated increase in the T-type Ca^2+^ current and the nuclear translocation of NFAT [50, 51]. Horikawa YT et al. also showed that the ventricles of mice overexpressing caveolin-3 and subjected to transverse aortic constriction show increased cGMP levels, decreased NFAT nuclear translocation, and increased nuclear Akt phosphorylation, compared to those of control mice. In addition, incubation with caveolin-3 adenovirus promotes the expression of caveolin-3 and ANP and the phosphorylation of Akt in cardiac myocytes [51]. According to Taniguchi T et al., [52] the loss of PTRF/cavin-1 protein expression is sufficient to induce a molecular program that leads to cardiomyocyte hypertrophy and cardiomyopathy, which is partly attributable to caveolin-3 reduction in the heart. Moreover, according to Lei et al. [53], caveolin-3 has been shown to exert a cardio-protective function against hyperglycemia-induced cardiac function; furthermore, hyperglycemia-induced excessive PKCβ_2_ activation reduces caveolin-3 expression and subsequently reduces Akt signaling, detrimentally affecting cardiac remodeling and function. Ca^2+^ disturbances due to reduced caveolin-3 also contribute to diabetic cardiomyopathy. According to Murfitt L et al., the loss of cavelion-3 might lead to increased nitrosylation of the ryanodine receptor by decreasing the inhibitory effect of caveolin-3 on eNOS, thus indicating another mechanism underlying the protective effect of caveolin-3 on diabetic cardiomyopathy [54]. Caveolin-1/caveolin-3-double knockout mice develop severe cardiomyopathy, compared to that in caveolin-1-knockout, caveolin-3-knockout, or wild-type mice [55]. Collectively, the cardiac-specific overexpression of caveolin-1 or caveolin-3 is a novel strategy to attenuate cardiac hypertrophy and heart failure [42, 51].

## 6. Caveolin and pulmonary hypertension

The pathophysiological mechanisms of pulmonary hypertension include endothelial dysfunction, impaired vasoconstriction, the remodeling of pulmonary microvessels, and intravascular thrombosis. Evidence suggests that caveolin-1 is a critical regulator of the pulmonary vascular function in animals and humans through the regulation of Ca^2+^ signaling [56] and eNOS activation [57]. However, the role of caveolin-1 in pulmonary hypertension varies in different vascular cells. Endothelial dysfunction, which is characterized by inflammation and oxidative stress, is the first step of pulmonary vascular wall damage. Endothelial injury caused by proinflammatory mediators leads to caveolin-1 depletion and eNOS uncoupling, consequently priming endothelial cells into a proinflammatory phenotype linked to oxidative stress-mediated BMPRII reduction; this is accompanied by elevated TGF-β, which promotes pulmonary vascular remodeling [58]. As a result, pulmonary hypertension occurs due to hyperactive eNOS and subsequent tyrosine nitration-dependent impaired PKG activity [59]. Wunderlich C et al. [57] found that caveolin-1 knockout mice exhibit enhanced oxidative stress due to hyperactivation of the nitric oxide pathway, which is significantly attenuated by the chronic inhibition of eNOS. They also showed that tetrahydrobiopterin (BH4) supplementation restores the normal BH4/BH2 ratio, thus reducing oxidant stress, and attenuating the dysfunction of endothelial and pulmonary hypertension [60].

Activation of the proliferative and anti-apoptotic pathways, due to the loss of the inhibitory function of endothelial caveolin-1, leads to vascular remodeling and pulmonary hypertension. According to Huang J et al. [61], monocrotaline-induced rat models of pulmonary hypertension exhibit the progressive loss of endothelial caveolin-1 due to extensive endothelial damage. Similarly, Zhao et al. [62] reported that caveolin-1-deficient mice show pulmonary hypertension and right ventricular hypertrophy. In their study, both right ventricular contractility and the diastolic function of the right ventricle decreased at baseline in caveolin-1^-/-^ mice, compared to that in wild-type mice. The author further showed that caveolin-1^-/-^ mice exhibit increased pulmonary vascular resistance associated with pulmonary vascular remodeling, which is characterized by increased medial thickness and the muscularization of distal pulmonary vessels [59]. Further, increased NO production in caveolin-1-knockout mice occurs to compensate for severe pulmonary vascular remodeling [63].

During pulmonary hypertension, in contrast to the activity of caveolin-1 in endothelial cells, the expression of caveolin-1 is increased and Ca^2+^ regulation is altered in vascular smooth muscle cells [64]. In a study by Huang J et al. [61], it was determined that the increased expression and activity of MMP-2 coupled with the enhanced expression of caveolin-1 in smooth muscle cells, subsequent to endothelial caveolin-1 deficiency, might facilitate cell proliferation and migration. Mu et al. [65] explained the role of caveolin-1 in smooth muscle cells in the progression of pulmonary hypertension. Their results suggested that increased expression of caveolin-1 in the aorta of pulmonary hypertensive rats affects the function of receptor-operated Ca^2+^ channels; this represents a mechanism of pulmonary vascular smooth muscle remodeling. Collectively, the different roles of caveolin-1 in pulmonary hypertension partly depend on the function of supporting cells.

**Figure 2.**
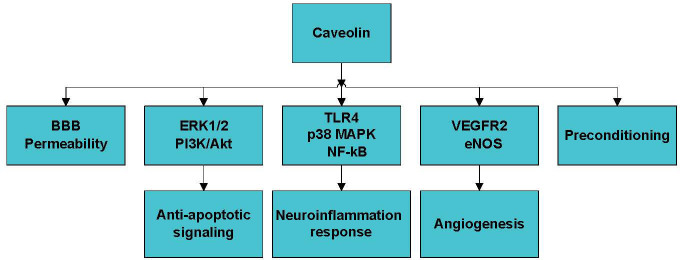
The mechanism of the role of caveolin in stroke. Caveolin plays a pivotal role in stroke by modulating BBB permeability, anti-apoptotic signaling, neuroinflammation, angiogenesis, and ischemic preconditioning. BBB, brain blood barrier.

## 7. Caveolin and stroke

Caveolin-1 also modulates the blood-brain barrier (BBB) permeability, pro-survival signaling, angiogenesis, neuroinflammation, and ischemic preconditioning, which are critical processes during stroke development (Fig. 2). Knowland D et al. [66] found that BBB disruption in response to ischemia is initiated by the upregulation of endothelial transcytosis, which is mediated by the overexpression of caveolin-1 in the early phase of reperfusion (6 h) and followed by the major remodeling of tight junction complexes in the late phase (24-48 h). This suggests that the stepwise impairment of transcellular, followed by paracellular, barrier mechanisms is responsible for BBB dysfunction during ischemic stroke. However, the authors also observed that caveolin-1^-/-^ mice exhibit increased stroke areas compared to those in wild-type mice. Therefore, this mechanism requires further investigation. According to Zhang S et al. [67], exposure to high glucose at the early reperfusion stage disrupts the BBB, which is associated with caveolin-1-mediated intracellular translocation and the autophagy lysosome-mediated degradation of ZO-1 protein.

The MAPK and phosphoinositide 3-kinase/protein kinase B (PI3k/Akt) pathways, which mediate apoptosis and autophagy, are also affected by caveolin-1 in the context of ischemia and reperfusion injury. ERK1/2, a major branch of the MAPK pathway, is associated with neuronal survival, and it protects against ischemic stroke via anti-apoptotic signaling. Yun JH et al. [68] showed that caveolin-1 exerts a positive effect on Src homology 2-containing protein tyrosine phosphatase 2 (SHP-2), which has been shown to positively modulate ERK1/2 activation; in contrast, the downregulation of caveolin-1 inhibits SHP-2 phosphorylation. Ischemic neuronal damage in mice is also augmented due to caveolin-1 deficiency and the subsequent reduction in p-ERK1/2 in mice [69]. However, the disparate effects of caveolin deficiency on ERK activation in neuronal versus cardiac cells remains unclear. The distributions of different tissues might contribute to these discrepant effects. Caveolin, which enhances the PI3K/Akt pathway, also exerts neuroprotective effects by promoting cell survival [70]. It has been established that inflammation plays a pivotal role in ischemic stroke. According to Niesman IR et al. [71], caveolin-1- and caveolin-3-knockout mice show increased levels of proinflammatory cytokines including IL-1β, IL-2, IL-6, IL-9, IL-10, IL-17, keratinocyte chemoattractant, monocyte chemotactic protein-1, and macrophage inflammatory protein-1α, ultimately leading to larger brain lesions. Alternatively, Wang et al. found that the overexpression of caveoin-1 attenuates proinflammatory cytokine (TNF-α and IL-6) production and increases anti-inflammatory cytokine (IL-10) production in the mouse macrophage cell line RAW264.7 by increasing p38 MAPK phosphorylation [72]. Furthermore, caveolin exerts anti-inflammatory effects by inhibiting NF-kB via the binding to TLR4, an upstream effector of the NF-kB pathway [73]. Angiogenesis is essential for neural recovery after ischemic stroke. A recent study by Chen et al. [74] found that exercise improves angiogenesis through the caveolin-1/VEGF pathway. Taken together, BBB permeability, cell survival, inflammation, and angiogenesis are involved in the regulation of ischemic stroke by caveolin.

## 8. Perspectives

### 8.1 Crosstalk between caveolin and autophagy

The crosstalk between caveolin and autophagy during the regulation of cell survival is now attracting great attention with respect to the progression of various diseases [33, 75, 76]. Caveolin-1 is known to regulate the cell cycle and apoptosis by modulating Ca^2+^ entry. Autophagy, a form of non-apoptotic cell death that is tightly regulated by more than 30 highly-conserved autophagy-related genes, is essential to maintain cell homeostasis and occurs through the release of nutrients from macromolecules and the elimination of unwanted proteins and cell organelles. Autophagy is a double-edged sword in cardiovascular diseases. Moderate autophagy exerts cardio-protective effects through its essential role in cell survival, whereas excessive autophagy has detrimental effects by triggering cell death in pathological settings.

In lipid metabolism homeostasis, autophagy regulates lipid efflux via ABCA1 and ABCG1. Both proteins facilitate macrophage reverse cholesterol transport and reduce foam cell formation. According to Lin et al., the molecular interaction between caveolin-1 and ABCA1 is associated with the HDL-mediated cholesterol efflux pathway in aortic endothelial cells [77]. Similarly, Gu et al. [78] identified an interaction between caveolin-1 and ABCG1 and elucidated an important role for caveolin-1 in ABCG1-mediated reverse cholesterol transport in HEK293 cells and cholesterol efflux to reconstituted HDL in THP-1-derived macrophages. Therefore, these findings suggest that caveolin-1 regulates ABCA1 and ABCG1 to modulate cholesterol homeostasis in macrophages. Oxy-LDL increases the translocation of NF-kB to the nucleus by upregulating caveolin-1, and ultimately attenuates protective autophagy and increases apoptosis in endothelial cells, thereby contributing to the progression of atherosclerosis [79].

It has been established that oxidative stress induces autophagy, and that autophagy in turn contributes to clearing irreversibly oxidized biomolecules. According to Takashi Shiroto et al. [80], the siRNA-mediated knockdown of caveolin-1 leads to a striking increase in expression of the cellular autophagic marker protein LC3BII in endothelial cells. In their study, the treatment of control or caveolin-1 siRNA-transfected cells with the lysosomal inhibitor bafilomycin A1 markedly increased LC3BII expression, indicating that the increase in LC3BII expression induced by caveolin-1 knockdown is due to increased autophagy flux and not a blockade in the terminal stages of the autophagy pathway. The investigators concluded that autophagy activation following caveolin-1 knockdown might reflect an adaptive response to the marked increase in oxidative stress caused by decreased caveolin-1 protein expression in endothelial cells [80]. However, Nah J et al. [81] revealed that the loss of caveolin-1 impairs autophagy and increases the infarct area following cerebral ischemia. Phosphorylated caveolin-1 at tyrosine-14 protects against cerebral ischemic damage by activating autophagy through its binding to the Beclin-1/VPS34 complex under conditions of oxidative stress [82]. According to Kassan A et al. [83], in the heart stimulated by ischemia and ischemia-reperfusion, caveolin-3-knockdown HL-1 cells (a cardiac muscle cell line) show decreased expression of autophagy markers (beclin-1 and LC3II), increased cell death, and increased levels of apoptotic proteins (cleaved caspase-3 and cytochrome c). Alternatively, caveolin-3-overexpressing cells increased autophagy markers expression, which indicates preserved mitochondrial function. Taken together, the role of caveolin in the regulation of autophagy and apoptosis in various different organs and cells suggests that caveolin-1 might be cell-type and protein-specific. Mechanistically, the different effects of caveolin on the PI3K/Akt pathway, a key pathway in the regulation of autophagy, could mediate this specificity.

### 8.2 Therapeutic promising

Despite the inhibitory effect of caveolin-1 on eNOS, a non-inhibitory and mutated caveolin-1 (Cav1 ^F92A^), produced through a substitution of alanine for phenylalanine, was found to increase eNOS expression and NO production [84]. Cav1^F92A^ inhibits the proliferation of smooth muscle cells in pulmonary vessels [84]. Mesenchymal stem cells are also an attractive option for the management of cardiovascular diseases. Yu et al. [85] revealed that Cav1^F92A^-modified rat bone marrow mesenchymal stem cells (rBMSCs/Cav1 F92A) activate the NO/cGMP pathway, inhibit TNF-α, TGF-β1, thrombospondin-1, and MGP expression, and consequently suppress cell migration in monocrotaline-treated human pulmonary artery smooth muscle cells (HPASMCs), suggesting that rBMSCs/Cav1 ^F92A^ might represent a therapeutic approach for pulmonary hypertension, which functions by inhibiting the switching of HPASMCs from a contractile to synthetic phenotype. According to Hiromura M et al. [86], the dipeptidyl peptidase (DPP)-4 inhibitor teneligliptin competes with CD26 in terms of its binding to caveolin-1, indicating that caveolin-1 is targeted by DPP-4 inhibitors to suppress TLR4-mediated inflammation in mouse and human macrophages, implying that it is a therapeutic target for atherosclerosis. Fasudi, which is commonly used to improve microvascular dysfunction, has been shown to exert protective effects on streptozotocin-induced diabetic nephropathy by blocking the VEGFR2/Src/caveolin-1 signaling pathway, suggesting that caveolin-1 could be targeted to ameliorate microvascular dysfunction [87]. It has also been showed that the autophagic protein LC3B exerts protective effects during the pathogenesis of pulmonary hypertension [88]. Wu et al. [41] found that a decrease in caveolin-1 due to the activation of Apelin-13/APJ leads to autophagy and subsequent cardiac hypertrophy. Taken together, the crosstalk between caveolin and autophagy potentially serves as a promising target for cardiovascular disease.

Traditional Chinese herbal medicines have been shown to be beneficial for the treatment of cardiovascular diseases by targeting caveolin. The Tiaopi Huxin recipe, a traditional Chinese medicine widely used to treat coronary heart disease, was recently shown to decrease the expression of caveolin-1, thereby improving endothelial function and reducing atheromatous lesions in ApoE^-/-^ mice [19]. Further, Yan et al. [89] showed that Zhuanggu Jianxi Decoction, a traditional Chinese medicine, inhibits IL-1β-induced activation of the caveolin-p38MAPK pathway, thereby suppressing the inflammatory response. Xuezhikang, the extract of red yeast rice, upregulates eNOS expression in the vascular endothelium, increases plasma NOx levels, and improves abnormal hemorheology in high cholesterol diet-induced atherosclerotic rats. Quercetin, a flavonol-type flavonoid derived from fruits and vegetables, exerts an anti-atherosclerotic effect by inhibiting caveolin-1 expression in endothelial cells [90]. Resveratrol, a polyphenolic compound present in red wine, exerts anti-oxidative and anti-inflammatory effects; it increases myocardial function and increases glucose transporter (GLUT-4) translocation to caveolar lipid raft fractions [91]. Resveratrol also increases the expression of caveolin-3, which is reduced in the diabetic myocardium [91]. Further, resveratrol-induced increases in the expression of caveolin-3 were found to be necessary for the activation of PI3K and Akt, which are upstream of the autophagy pathway [92]. Taken together, traditional Chinese medicines can potentially attenuate cardiovascular disorders by regulating the crosstalk between caveolin and autophagy.
